# Using Bones to Shape Stones: MIS 9 Bone Retouchers at Both Edges of the Mediterranean Sea

**DOI:** 10.1371/journal.pone.0076780

**Published:** 2013-10-11

**Authors:** Ruth Blasco, Jordi Rosell, Felipe Cuartero, Josep Fernández Peris, Avi Gopher, Ran Barkai

**Affiliations:** 1 The Gibraltar Museum, Gibraltar, Gibraltar; 2 Àrea de Prehistoria, Universitat Rovira i Virgili (URV), Tarragona, Spain; 3 IPHES, Institut Català de Paleoecologia Humana i Evolució Social, Tarragona, Spain; 4 Departamento de Prehistoria y Arqueología, Universidad Autónoma de Madrid, Madrid, Spain; 5 Servei d’Investigació Prehistòrica (SIP), Museo de Prehistoria, Diputación de Valencia, Valencia, Spain; 6 Institute of Archaeology, Tel Aviv University, Tel Aviv, Israel; University of Oxford, United Kingdom

## Abstract

A significant challenge in Prehistory is to understand the mechanisms involved in the behavioural evolution of human groups. The degree of technological and cultural development of prehistoric groups is assessed mainly through stone tools. However, other elements can provide valuable information as well. This paper presents two bone retouchers dated to the Middle Pleistocene MIS 9 used for the shaping of lithic artefacts. Originating from Bolomor Cave (Spain) and Qesem Cave (Israel), these two bone retouchers are among the earliest of the Old World. Although the emergence of such tools might be found in the latest phases of the Acheulean, their widespread use seems to coincide with independently emergent post-Acheulean cultural complexes at both ends of the Mediterranean Sea: the post-Acheulean/pre-Mousterian of Western Europe and the Acheulo Yabrudian Cultural Complex of the Levant. Both entities seem to reflect convergent processes that may be viewed in a wider cultural context as reflecting new technology-related behavioural patterns as well as new perceptions in stone tool manufacturing.

## Introduction

Meat consumption is deeply rooted in human existence and seems to have been essential as early as the appearance of the genus *Homo* [1]. Animal bones accumulated in the earliest archaeological sites of Africa show anthropogenic damage resulting from nutritional purposes - i.e., defleshing cut-marks and fractures of marrow extraction. While such bone remains were available as potential raw material [2], bones did not partake in the manufacturing of tools until well into the Acheulean cultural complex (1.5−0.4/0.2 Mya), when they were prevalently used as both raw material to be shaped or as tools for shaping. Large mammal bones, especially those of elephants, were used for making flakes and tools, including objects that resemble the most characteristic Acheulean stone handaxes [3]–[12]. Deer antlers, and more rarely joints (epiphyses of limb bones), have been also used in knapping large Acheulean flint tools, mostly handaxes [13]–[14], e.g., in the case of the UK site of Boxgrove (around 0.5 Mya) where antlers and a distal epiphysis of a red deer humerus have been used as percussors [15]–[16]. This last case was identified through pitting found on the distal articular surface with small lithic fragments embedded within [16]. While in fresh state, these elements are sufficiently hard and heavy to be used as hammers for knapping Acheulean handaxes, the most common elements in subsequent periods are related to mid-shaft fragments that were recycled pursuant breakage for marrow consumption. These bones were used to shape lithic tools by percussion or pressure applied on the edges of stone flakes. These activities often generate short incisions arranged transversely or obliquely on the cortical surface of the bone. These striations are deep and with a V-shaped bottom, composed of a right angle next to another more acute in cross section, similar to chop-marks [17]. They often appear clustered in specific areas of the bone, called active areas. Depending on the intensity of use, some activity marks may overlap, forming deep pits (e.g., [18]–[25]). Such bones are commonly found in post-Acheulean contexts, mainly in European Middle and Upper Palaeolithic assemblages [25] but not so in Lower Palaeolithic sites of the Acheulean cultural complex in Africa, Asia, or Europe. Six bone retouchers were recovered from layers 7 and 6 of Orgnac 3 (France) dated to MIS 9, which are suggested to be associated with the final phases of the Acheulean in this site [26]. For Moncel et al. [26], the absence of the Levallois technique in these layers is related to the continuity of the Upper Acheulean rather than with the development of Middle Palaeolithic techno-complexes. In spite of this, the habit of using bone fragments of consumed animals as retouchers for shaping stone tools seems to have emerged and adopted routinely only after ca. two million years of meat consumption, and especially with the development of post-Acheulean cultural entities. Collectively viewed, these retouchers seem to be part of the significant behavioural changes that took place between 400 and 300 kya ago in different parts of the Old World.

### The Archaeological Contexts and Dating

#### Bolomor Cave

**Figure 1:**
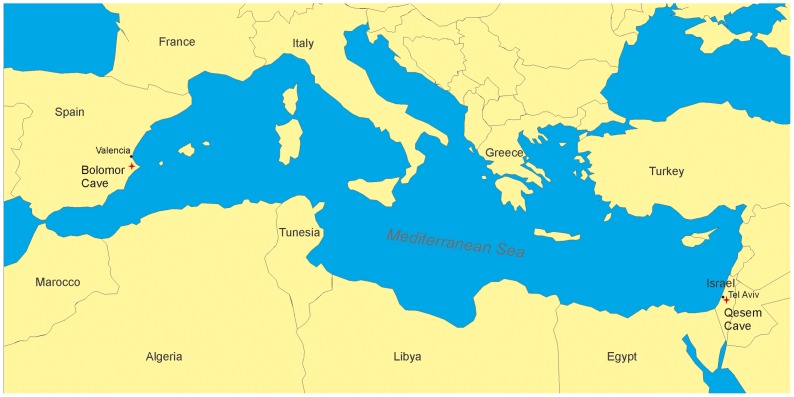
Location of Qesem Cave (Israel) and Bolomor Cave (Spain).

Bolomor Cave is located in Valencia (Spain) at 100 m a.s.l. (Figure 1). Its stratigraphic sequence is divided into 17 levels. The bottom levels were dated by amino acid racemisation (AAR) to 525±125 kya (level XVIIa) and by U/Th to >350 kya (level XVb). However, the study of the magnetic susceptibility of the sediment studied by B.B. Ellwood in Fernández Peris [27] showed a warm period at the beginning of the stratigraphic sequence, setting the bottom deposit within MIS 9. This younger age coincides with palaeoclimatic and chronological data obtained by means of sedimentological and paleontological analysis carried out in the cave [27]–[30]. The thermoluminescence (TL) dates from level XIV range from 233±35 kya to 225±34 kya. AAR dating of level XIIIc yields an age of 228±53 kya and TL dating yielded the age of 152±23 kya for level XIIIa and 121±18 kya for level II. A wide range of animal species were processed and consumed by the human groups of Bolomor, including large and small ungulates as well as smaller taxa such as lagomorphs, tortoises, and birds [31]–[33]. The bone retoucher presented here was recovered at sublevel XVIIa. This sublevel contains 1732 faunal remains, of which 1016 have been identified at taxonomical level. This record includes 12 species of large and small ungulates with a predominance of *Cervus elaphus*, *Equus ferus* and *Oryctolagus cuniculus*, and 4 remains belonging to *Canis* cf. *lupus*. The proportion of long bone fragments (n = 677; 39.1%) is higher than flat bones (n = 317; 18.3%). Among the long bones, mid-shaft fragments show a significant representation (n = 406 of 677; 60%). Anthropogenic evidence caused during bone breakage has been documented on 117 ungulate remains (6.8%) in form of percussion notches, impact flakes and peeling [32]–[33]. The lithic technology is characterized by flake production, which is not typologically related to the peninsular Acheulean, and by lithic recycling. The lithic assemblage from level XVII shows a predominance of flakes, a scarce presence of the Levallois technique, and a predominance of denticulates and scrapers. Retouched artefacts show denticulate forms in 53.5% of the cases, followed by squamous (39.4%) and scalariform (7%) formats [27]. No handaxes were found and therefore, the Bolomor assemblages can be assigned to an early post-Acheulean industry developed in Western Europe during the second half of the Middle Pleistocene. Bolomor Cave shows clear evidence of habitual use of fire documented from as early as level XIII (MIS 7c) [34]. Human remains from Bolomor are compatible with European Middle Pleistocene hominin fossils, and comprise a small sample in which dental elements stand out in comparison with postcranial elements [35].

#### Qesem Cave

Qesem Cave is situated 12 km east of the Mediterranean coast in a hilly limestone terrain 90 m.a.s.l. (Figure 1). The cave is a sediment-filled chamber estimated at ∼20×15 m in size and ∼10 m high with an additional, recently uncovered deeper chamber yet to be excavated. Ongoing excavation has exposed ∼9.5 m of deposits containing sediments of natural and anthropogenic origins. The stratigraphic sequence is divided in two: a lower part (∼5.0 m thick) comprising sediments with clastic contents and gravels, and an upper part (∼4.5 m thick) mostly comprising cemented sediments with a substantial ashy component. As indicated by the presence of ashes, burned flint [36], and abundant burned bones [37], the habitual use of fire is attested throughout the sequence and micromorphological studies indicate that fire was possibly more intensively used in the upper part of the Qesem Cave sequence [38]. The entire sequence of Qesem Cave is assigned to the late 420–200 kya years old Lower Palaeolithic Acheulo Yabrudian Cultural Complex (AYCC) [39], post-dating the Acheulean and pre-dating the Mousterian. The faunal assemblages are dominated by fallow deer complemented by other species such as aurochs, horse, wild pig, red deer, and tortoise. Not all body parts are present, indicating that carcasses were first processed off-site and only selected parts were brought to the cave. Cut-marks and traces of burning were found on quite an impressive number of bones [37] indicating butchering and marrow extraction. The bone retoucher presented here comes from the lower stratigraphic sequence, where 1326 faunal remains were identified (Unit III in Stiner et al. [37], [40]). The majority of these corresponds to fragments of limb bone shafts and head parts attributed to medium and small-sized ungulates with a relevant predominance of *Dama* cf. *mesopotamica* and in a lesser extent, of *Cervus elaphus*, *Equus ferus*, *Bos primigenius*, *Sus scrofa* and *Testudo* cf. *graeca*. Cone (percussion) fractures were recognized on 19% of bones from Unit III [37]. The lithic assemblages of the cave are dominated by the AYCC Amudian blade industry [41]–[42]. The AYCC Yabrudian scraper-dominated industry appears at Qesem Cave in three stratigraphically and spatially distinct areas [41]. An Amudian, blade-dominated assemblage composed of 2560 artefacts from the close vicinity of the bone retoucher was analyzed and published in detail [43]. This assemblage includes 380 retouched items, characterized by a striking dominance of retouched and backed blades (62.6% of the shaped items), while retouched flakes (11.5%) and side and end scrapers appear in small quantities (6% and 3% respectively). In addition, flint recycling was systematically practiced in both industries. A study of human dental remains [44] concluded that the hominins inhabiting Qesem Cave were not *H. erectus* but rather similar to later modern populations (e.g., Skhul/Qafzeh) of this region, with some Neanderthal affinities as well. These cultural and biological transformations might indicate the emergence of a new hominin lineage in the Middle Pleistocene Levant [45].

## Data Presentation: Bone Retouchers

### Bolomor Cave: CB XVIIa C4’/126; Z = 914

**Figure 2:**
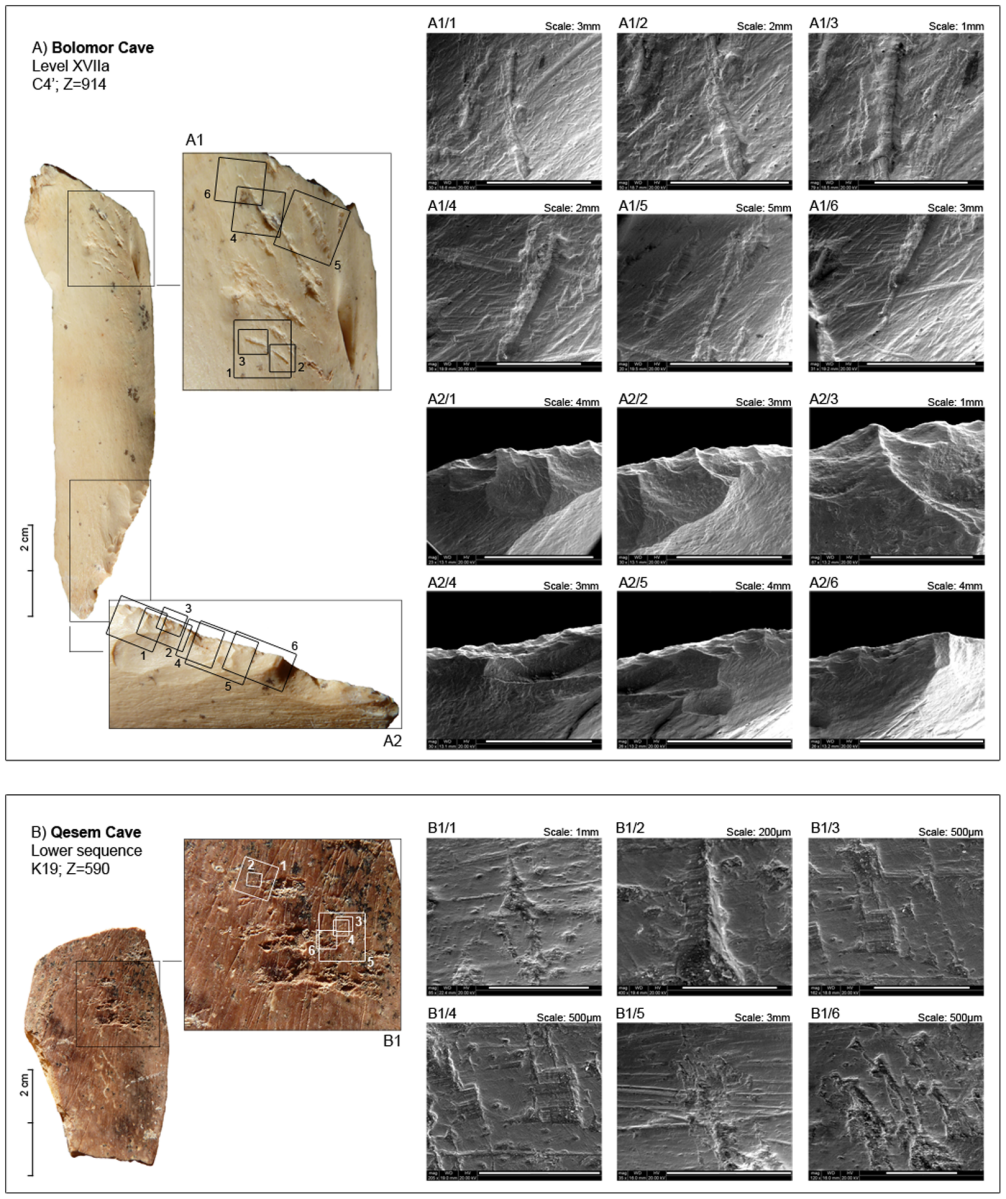
Macroscopic and microscopic view (ESEM) of bone retouchers from Bolomor Cave, Spain (A) and Qesem Cave, Israel (B).

This bone is a shaft fragment of the right femur (lateral and anterior side) of a red deer (*Cervus elaphus*) (86.8×20.8×4.1 mm; Figure 2A). Its breakage planes show curved V-shaped outlines, oblique angles, and smooth edges, all of which indicate the fresh state of the bone when it was fractured [46]. The identical colour and patina of both fractures and cortical surface of the fragment indicate that the breakage occurred prior to excavation and not as a result of it. The specimen displays no evidence of significant mechanical or chemical alteration, and its well-preserved state allows for the identification of a discrete concentration of oblique, short, and deep striations on the proximal metadiaphysis (active zone). At a microscopic level, the V-shaped bottom of the striations is composed of a right angle set next to another, more acute angle. These characteristics are in contrast with morphology and delineation criteria commonly used to identify cut marks [47]–[49]. However, they are similar to damage identified during retouching activities which has been described both experimentally and archaeologically by several researchers [23], [25]. In addition to this damage, long and continuous parallel striae, perpendicular to the percussion pits or main striations (thus parallel to the major axis of the fragment), can be observed. To interpret these striae, we must take into account that to create a continuous retouch for a stone scraper necessitates the use of retouchers with some specific features: a flat or slightly convex, broad surface, used with frontal percussion against the edge of the flake, which was used as support. Both diaphysis fragments and small flat pebbles could have been used for this task. When the angle of the retouched edge was close to 90°, no other manipulation would have been necessary. However, when the retouched edge was sharp, a second type of manipulation would be necessary: scratching. This action could have been accomplished with a different object (a small pebble) or with the same retoucher on the diaphysis, by simply performing tangential abrasion of the edge. These two actions would have resulted in the type of striae found on the diaphyseal fragment, used as a retoucher at Bolomor XVIIa, which presents both percussion pits and long, parallel, scratched striae (Figure 2, A1/4). Finally, this fragment presents a series of overlapping planes and continuous retouches along the distal edge opposite the active region. The angles of these cortical removals are planar or semiplanar but continuous and deep whilst on the distal segment, most likely due to re-sharpening.

### Qesem Cave: CQ’01 Lower Sequence K19; Z = 590

This bone corresponds to a long bone shaft of a medium-sized animal (43.5×25.8×6.6 mm; Figure 2B). It presents five breakage planes with: 1) transverse, longitudinal and curved outlines; 2) right and mixed angles; and 3) smooth and jagged surface edges [46]. Bone fractures have the same colour and patina as the cortical bone surface, indicating that the breakage was generated at or near the time of deposition. Only one plane located at the right edge of bone displays a lighter colour, suggesting it reflects a new break produced during excavation. The cortical side is well preserved and, as in the case of Bolomor, shows no evidence of significant mechanical or chemical damage that would hamper the observation of anthropogenic modifications. The fragment displays damage typically generated by use of bones as soft retouchers. The marks are located at the upper centre of the cortical surface and are configured in short, deep, closely clustered, overlapping pits associated with thin, elongated striations. These striae are oriented perpendicular to the long axis of the fragment. Microscopic analysis discloses that these V-shaped striations are generally asymmetrical in cross-section. In addition to retoucher marks, the cortical surface presents a long band of parallel striations, longitudinal and oblique to the main axis of the fragment, which are interrupted by traces left by the use of the fragment as a retoucher implicating that they were generated during previous activity. This band of marks seems to have been produced by scraping of the bone to remove the periosteum, either for nutritional purposes (no relationship with operative chain of production) or to clean its surface before using it as a retoucher (preparatory scraping before flint working).

## Discussion and Conclusions

Analyses of bone retouchers have provided contradictory results regarding the question whether hominids selected specific bone blanks for the task. Some authors have proposed that no apparent selection was made for particular bone types, animal taxa, or fragment sizes [20] whereas others have suggested a tendency towards selected skeletal elements [25], [50]. While selection and use criteria of these fragments are not clear, absent or scarce preparation could indicate purely morphological patterns. In the case of Bolomor, the bone retoucher has been intentionally shaped at the edge opposite the active area. The non-invasive character of this shaping seems to have left the original morphology of the bone quite unchanged. The morphological characteristics of the single bone retoucher recovered at sublevel XVIIa of Bolomor accord well with the selection pattern of bone fragments in the later Middle Palaeolithic site of Noisetier Cave [25] as well as in Payre, France [51], where the longest and thickest diaphysis fragments were preferred. Some experimental work indicates that these bones were used to retouch the sharp edges of stone flakes while fresh [25], [51]–[52]. The higher weight (or density) of fresh bone would have facilitated the shaping of lithic objects (mainly by low angle retouch), generating clustered and overlapping marks of variable depths on well-defined areas [53]. In contrast, dry bone retouchers show a higher number of pits and greater loss of cortical tissue, producing exfoliation traces similar to weathering processes [54].

Although it is difficult to evaluate the state of freshness of the bone blank at the time of its use, the bone retoucher from Bolomor seems to correspond to the use of a fresh, defatted bone, the elasticity of which was still intact. The resulting zone can be qualified as discrete (used for a short time) because the striations are well-defined, isolated and not associated with a loss of cortical tissue. In contrast, the bone retoucher from Qesem Cave displays a slight increase of pits, scores, and exfoliation in the very well-defined active area. This type of modification seems to be consistent with the employment of a semi-fresh bone. Another difference between the two retouchers is the presence of scraping-marks. All flesh had to be removed from bone blanks, and several authors have proposed that for hammer and retoucher efficiency, it was also necessary to remove the periosteum [20], [51]. According to Tartar ([55], p.133), retouchers with no traces of preparatory scraping would have been used once the periosteum was dry, when it no longer presented an obstacle for use. The Qesem bone retoucher shows scraping incisions aimed at removing the periosteum. This fact can be related to the nutritional processing of the carcass or the preparation of the bone for use as a retoucher. Otherwise, using a semi-fresh (i.e., partially dried) bone as a retoucher would have dissociated the scraping stage (and marks) from the lithic chain of operation. In the case of Bolomor, no marks have been identified that could be associated with the removal of the periosteum. This fact does not imply, however, that the membrane remained adhered to the bone while it was used as a retoucher, as it might have been removed by means of pulling or by a combination of scraping and pulling in previous processes of bone breakage.

The two bone retouchers presented herein originating at these two sites respectively are of the few earliest examples of such tools known to date and although early they both possess typical morphological and functional characteristics of such tools. Despite the distance between sites, the use of bone as retoucher emerged in a similar chronology (MIS 9). Simultaneously, other European sites show similar objects, such as sublevel TD10-1 of Gran Dolina, Spain [53], level 7 and 6 of Orgnac 3 [26] and levels E and H of La Micoque, France [56]. Although bone hammers can be punctually found in previous moments, e.g. Boxgrove (MIS 13) [15], [16], this technical behaviour seems to have become widespread from MIS 9. From this time on, bone retouchers are commonly found in archaeological sites and they appear to have been used up to the Upper Palaeolithic.

Interconnections between the Spanish and Near Eastern post-Acheulean cultural complexes are not feasible, thus this similar technological (and cultural) advancement indicates possible convergent developments. Both of these disparate cultural complexes were innovative at their time, consisting of a series of newly introduced behaviours and qualities unknown from earlier Acheulean sites, such as the habitual use of fire, the presence of large amounts of burnt bones (roasting), and lithic recycling. In the case of bone retouchers, their appearance and generalized use seem to have marked a new manner of bone use possibly reflecting a novel view of discarded bone recycling and therefore, an innovative human behaviour. The employment of bone tools in achieving an end hitherto achieved by use of other raw materials is not merely a technological innovation. The introduction of bones that originated in hunted, defleshed, and consumed animals into the sphere of lithic production brings together the two basic elements of prehistoric life - stone tool making and animal hunting and consumption. Such an early integration of these two primordial defining elements of human Palaeolithic existence suggests cultural convergence. In the case of Bolomor and Qesem Cave it evokes thoughts on the reason why two such different and geographically remote entities, and probably also two different hominins, show such similar and simultaneous innovation.

## Method summary

The Qesem Cave project has a permit by the Antiquities Authority of Israel following the law of Antiquities of Israel issued annually since the year 2001. The field-work/research at Bolomor Cave (Valencia, Spain) is carried out in strict accordance with the Cultural Heritage law of Valencia. Fossil remains from Bolomor Cave are stored in the Prehistory Museum of Valencia under the authority of the Provincial Council of Valencia, Spain. Fossil remains from Qesem Cave are put in storage in the Department of Archaeology of the Tel-Aviv University, Israel. No permits were required for the described study, which complied with all relevant regulations.

Surface alterations were treated at both macroscopic and microscopic level. For microscopic study an Olympus Europe SZ11 (magnification up to 110) and ESEM (Environmental Scanning Electron Microscope, FEI QUANTA 600) were used. Percussion marks were identified according to criteria described by Spencer de Gruchy and Roberts [17] and compared with cut-marks, carnivore tooth-marks and geochemical etching [48], [57]–[58]. Following Villa and Mahieu [46], bone breakage was analyzed in terms of fracture outline, angle, and edge. Additionally, criteria as “old” or “new” were distinguished by colour changes in the fracture surface of bone fragments.
