# Correction: Using Bones to Shape Stones: MIS 9 Bone Retouchers at Both Edges of the Mediterranean Sea

**DOI:** 10.1371/annotation/347f8ee9-c35b-4e51-8f9a-a40ae34e634c

**Published:** 2014-01-17

**Authors:** Ruth Blasco, Jordi Rosell, Felipe Cuartero, Josep Fernández Peris, Avi Gopher, Ran Barkai

The third sentence in the second paragraph of the Methods summary should have cited several authors [20-25] instead of Spencer de Gruchy and Roberts [17]. 

The correct sentence should read as follows, "Percussion marks were identified according to criteria described by several authors [20-25] and compared with cut-marks, carnivore tooth-marks and geochemical etching [48,57-58]."

